# Imaging Dopaminergic Neurotransmission in Neurodegenerative Disorders

**DOI:** 10.2967/jnumed.121.263197

**Published:** 2022-06

**Authors:** Elon D. Wallert, Elsmarieke van de Giessen, Remco J.J. Knol, Martijn Beudel, Rob M.A. de Bie, Jan Booij

**Affiliations:** 1 Department of Radiology and Nuclear Medicine, Amsterdam UMC, University of Amsterdam, Amsterdam, The Netherlands;; 2 Department of Radiology and Nuclear Medicine, Amsterdam UMC, Vrije Universiteit, Amsterdam, The Netherlands;; 3 Department of Nuclear Medicine, Noordwest Ziekenhuisgroep, Alkmaar, The Netherlands; and; 4 Department of Neurology, Amsterdam UMC, University of Amsterdam, Amsterdam, The Netherlands

**Keywords:** dopamine, neurodegeneration, PET, SPECT, Parkinson

## Abstract

Imaging of dopaminergic transmission in neurodegenerative disorders such as Parkinson disease (PD) or dementia with Lewy bodies plays a major role in clinical practice and in clinical research. We here review the role of imaging of the nigrostriatal pathway, as well as of striatal receptors and dopamine release, in common neurodegenerative disorders in clinical practice and research. Imaging of the nigrostriatal pathway has a high diagnostic accuracy to detect nigrostriatal degeneration in disorders characterized by nigrostriatal degeneration, such as PD and dementia with Lewy bodies, and disorders of more clinical importance, namely in patients with clinically uncertain parkinsonism. Imaging of striatal dopamine D_2/3_ receptors is not recommended for the differential diagnosis of parkinsonian disorders in clinical practice anymore. Regarding research, recently the European Medicines Agency has qualified dopamine transporter imaging as an enrichment biomarker for clinical trials in early PD, which underlines the high diagnostic accuracy of this imaging tool and will be implemented in future trials. Also, imaging of the presynaptic dopaminergic system plays a major role in, for example, examining the extent of nigrostriatal degeneration in preclinical and premotor phases of neurodegenerative disorders and to examine subtypes of PD. Also, imaging of postsynaptic dopamine D_2/3_ receptors plays a role in studying, for example, the neuronal substrate of impulse control disorders in PD, as well as in measuring endogenous dopamine release to examine, for example, motor complications in the treatment of PD. Finally, novel MRI sequences as neuromelanin-sensitive MRI are promising new tools to study nigrostriatal degeneration in vivo.

Imaging of dopaminergic transmission in the brain is an important tool in neurodegenerative disorders such as Parkinson disease (PD) and dementia with Lewy bodies (DLB), not only as a research topic but also, frequently, for use in routine practice. In the first part of this review, we describe the role of dopaminergic imaging in routine practice. In the second part, we discuss its role in research.

## IMAGING BRAIN DOPAMINERGIC NEUROTRANSMISSION IN NEURODEGENERATIVE DISORDERS IN ROUTINE PRACTICE

### Imaging of Presynaptic Nigrostriatal Dopaminergic Pathway

In routine practice, imaging of the presynaptic nigrostriatal dopaminergic pathway is used to determine whether this is degenerated and, therefore, to differentiate patients with nigrostriatal degeneration from those without degeneration. The most common, and the relatively common, diseases characterized by nigrostriatal degeneration are PD, DLB, multiple-system atrophy (MSA), progressive supranuclear palsy (PSP), and corticobasal degeneration (1–3). PD, including PD dementia, and DLB are increasingly considered a disease continuum in view of their similar pathology (4). We will here focus on imaging of the dopaminergic system in these disorders.

**FIGURE 1.**
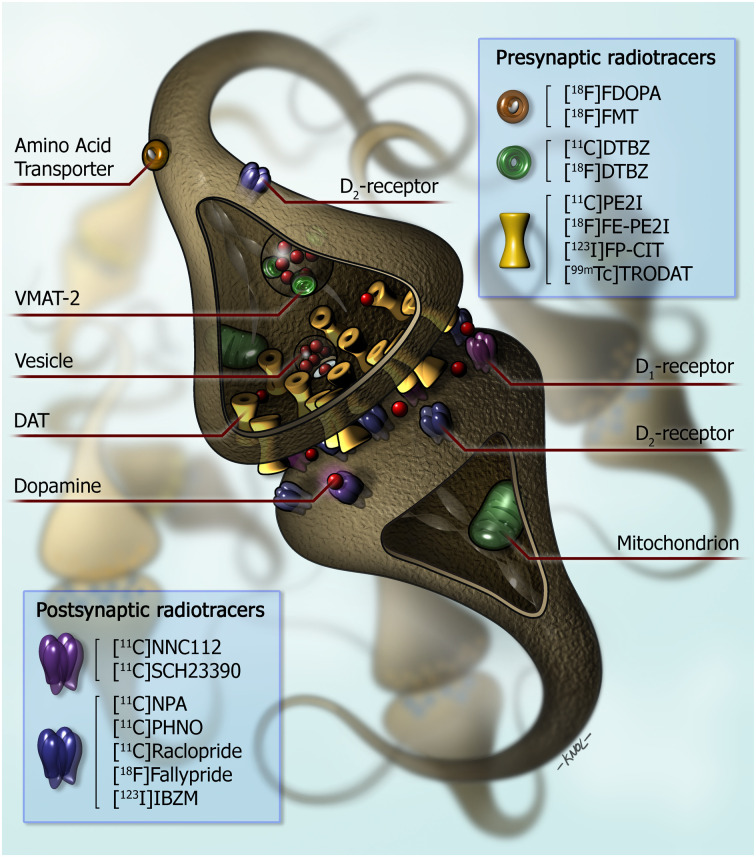
Simplified diagram of striatal dopaminergic synapse. On presynaptic side, markers for imaging of integrity of dopaminergic neurons in humans are shown. ^18^F-FDOPA and ^18^F-FMT PET provide measures of structural and biochemical integrity of dopaminergic neurons. ^11^C-DTBZ and ^18^F-DTBZ are radiopharmaceuticals for vesicular monoaminergic transporter. Substituted (nor)phenyltropanes (^11^C-PE2I, ^18^F-FE-PE2I, ^123^I-FP-CIT, and ^99m^Tc-TRODAT) are frequently used PET and SPECT radioligands for imaging of DAT. On postsynaptic side, ^11^C-NNC112 and ^11^C-SCH23390 radiopharmaceuticals for dopamine D_1_ receptor are shown. Dopamine D_2_ receptors are expressed predominantly on postsynaptic side as compared with presynaptic side of dopaminergic synapse. ^11^C-NPA and ^11^C-PHNO are agonist radioligands for dopamine D_2/3_ receptors. Commonly used antagonist radioligands for D_2/3_ receptors are substituted benzamides (^11^C-raclopride, ^11^C-FLB 457, ^18^F-fallypride, and ^123^I-IBZM). (Reprinted from (6).)

The nigrostriatal dopaminergic pathway can be imaged using radiopharmaceuticals for the dopamine transporter (DAT), for the vesicular monoamine transporter-2 (VMAT-2), or for aromatic L-amino-acid decarboxylase (AADC) activity (mainly using ^18^F-6-fluoro-l-dopa [^18^F-FDOPA]) (Fig. 1) (5). Regarding DAT imaging, many SPECT and PET tracers have been developed successfully (6). For VMAT-2 imaging, only PET tracers have been developed successfully (Fig. 1). Since the radiopharmaceutical ^123^I-labeled 2β-carbomethoxy-3β-(4-iodophenyl)-*N*-(3-fluoropropyl) nortropane (^123^I-FP-CIT, or ^123^I-ioflupane, commercialized as DaTscan [in the United States; GE Healthcare], DaTSCAN [in Europe; GE Healthcare], or Striascan [Curium]) is the only licensed radiotracer to image the nigrostriatal dopaminergic pathway by the Food and Drug Administration and European Medicines Agency, most hospitals and institutes use this radiopharmaceutical to assess the integrity of the nigrostriatal pathway in routine clinical studies.

#### DAT Imaging

**FIGURE 2.**
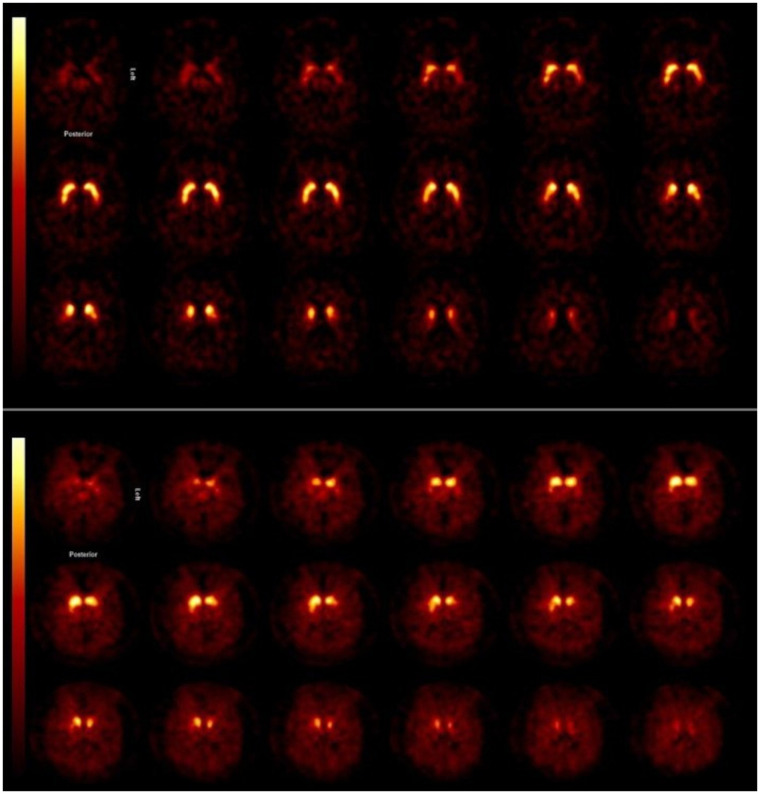
Transversal ^123^I-FP-CIT SPECT images obtained in patient with CUPS without striatal DAT loss (top) and in CUPS patient with striatal DAT loss (bottom). Asymmetric striatal binding can be seen, as well as severe loss of DAT binding, especially in putamen, in subject with dopaminergic deficit. This study was acquired on brain-dedicated SPECT system (InSPira; NeuroLogica).

In PD, the loss of striatal DAT binding is typically more pronounced in the putamen than in the caudate nucleus (6). Characteristically, the loss of DAT binding starts in the posterior part of the putamen and is more pronounced in the dorsal than in the ventral part of the putamen (Fig. 2). Also, commonly, binding of the DAT tracer is lower at the contralateral than the ipsilateral striatum (i.e., contralateral to the clinically most affected body side) (Fig. 2). The loss of striatal DAT binding in PD can already be detected in the early motor phases of the disease and even at the premotor and preclinical stage (7–10). In line with this fact, systematic reviews and metaanalyses showed that DAT imaging is a sensitive and specific imaging tool to detect nigrostriatal degeneration in PD (5*,*^11^). The diagnostic accuracy of DAT imaging is also high in patients with clinically uncertain parkinsonism (CUPS) and impacts clinical decision making, emphasizing its usefulness in clinical practice (11–14). This is of relevance since it can be challenging to diagnose PD clinically, especially in the early motor stage of disease (15*,*^16^). In addition to visual inspection, a quantitative or semiquantitative approach may increase reader confidence and create more reproducible reporting (17*,*^18^).

Clinically and neuropathologically, MSA can be divided into MSA with predominantly parkinsonian signs (MSA-P) or MSA with cerebellar features (MSA-C). As in PD, DAT binding is lower in the putamen than in the caudate nucleus (19). Some studies have demonstrated lower and more symmetric striatal DAT binding in MSA-P patients than in PD patients. However, the differences are relatively small, and findings consequently are inconsistent and cannot differentiate between these diagnoses at an individual level (20*,*^21^). Nevertheless, at the individual level there is a clear overlap in binding ratios between MSA-P and PD patients (20*,*^21^), thus precluding a role for DAT SPECT imaging in differentiating between degenerative parkinsonian diseases in daily clinical practice. DAT PET imaging offers the advantage of a better spatial resolution than DAT SPECT and, subsequently, a subregional analysis of striatal DAT binding. In this regard, Oh et al. showed that DAT PET imaging may indeed be able to differentiate PD from MSA-P at a group level (22). However, also in that study, MSA-P patients could not be differentiated completely from PD patients at an individual level.

In MSA-C, on average, striatal DAT binding is higher than in MSA-P and PD (19) and can even sometimes be normal (21*,*^23^). Fortunately, in routine practice, MSA-C patients can frequently be differentiated clinically from PD patients rather easily.

As in MSA-P and PD, DAT imaging is a sensitive means to detect loss of striatal DAT binding in PSP (21). Interestingly, a recent systematic review showed that striatal DAT binding in PSP is clearly lower than in PD and MSA-P (5). More specifically, DAT binding in PSP was on average approximately 34% and 18% lower than in PD in the caudate nucleus and putamen, respectively. Although studies on PSP, PD, and MSA-P do show an overlap in striatal DAT binding at an individual level, a very low DAT binding, particularly of the caudate nucleus, in an individual parkinsonian patient with a short disease duration (e.g., <2 y) might indicate the development of atypical parkinsonism. In corticobasal degeneration, the loss of striatal DAT binding can be very asymmetric, although it can also mimic the typical pattern of PD and can sometimes even be normal (24).

In Europe, ^123^I-FP-CIT SPECT is also frequently used to differentiate DLB from Alzheimer disease, since it is also approved by the European Medicines Agency for this indication. In Alzheimer disease, striatal DAT is typically not reduced, whereas DLB is characterized by loss of striatal DAT binding (25*,*^26^). Characteristically, in DLB, DAT binding is lower in the putamen than in the caudate nucleus; however, this posterior–anterior gradient may be more pronounced in PD than in DLB (27). Recent metaanalyses on the value of DAT imaging in DLB concluded that DAT imaging has a high diagnostic value for detecting DLB versus Alzheimer disease (sensitivity of 86.5% and specificity of 93%) and is more accurate than the clinical diagnosis (28*,*^29^). However, some recent studies suggested that DAT imaging may initially be normal in a relatively rare DLB subtype (∼10% of cases), with possibly a different severity or spread of α-synuclein pathology (neocortical predominant subtype) (30–32).

Among patients with clinically diagnosed PD who were enrolled in PD trials, around 10%–15% have been found to have normal DAT SPECT findings, also referred to as scans without evidence of dopaminergic deficit (33). Interestingly, in most patient with such scans, abnormal DAT SPECT scans do not develop on long-term follow up (11). In line with this observation, it is now well accepted that normal DAT SPECT findings exclude PD (34).

Although most institutes use ^123^I-FP-CIT SPECT to assess striatal DAT binding in routine practice, some institutes also use DAT PET tracers such as ^18^F-FE-PE2I (6).

#### AADC Imaging

**FIGURE 3.**
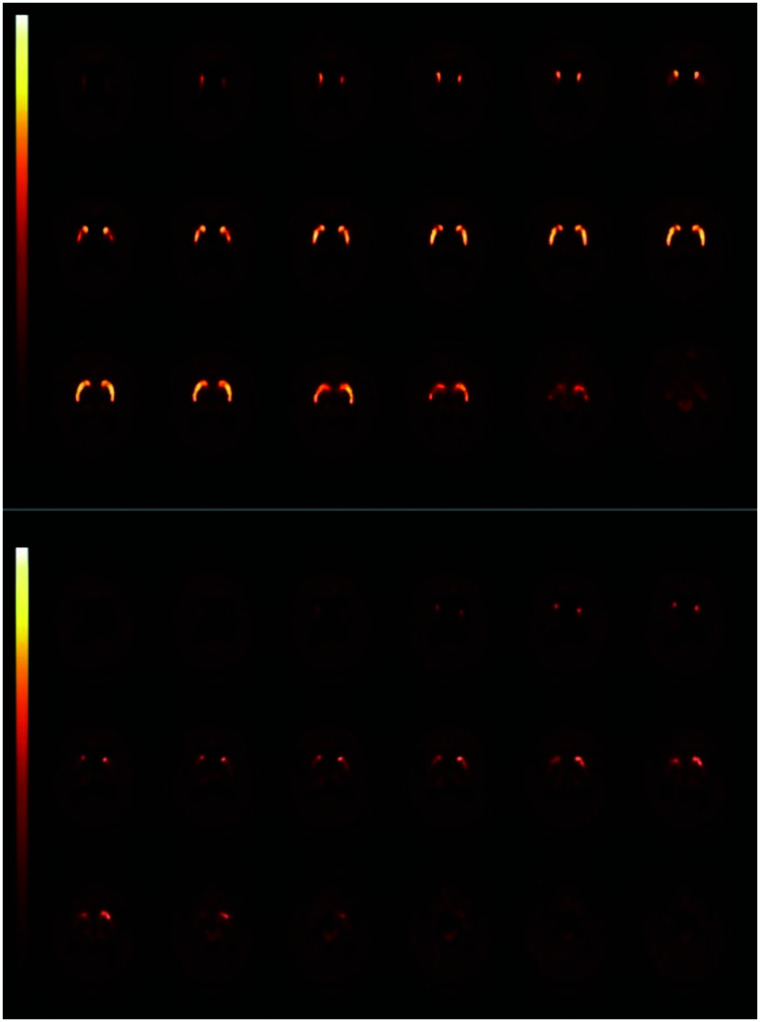
Transversal planes of ^18^F-FDOPA PET images obtained in patient with CUPS without nigrostriatal cell loss (top) and in CUPS patient with nigrostriatal degeneration (bottom). Asymmetric striatal uptake can be seen, as well as severe loss of ^18^F-FDOPA uptake, especially in putamen, of subject with nigrostriatal degeneration.

Many studies have assessed striatal AADC activity in neurodegenerative disorders, particularly PD, using ^18^F-FDOPA PET (5). The pattern loss of striatal AADC mimics the loss of striatal DAT binding in diseases such as PD, MSA-P and PSP (Fig. 3) (5*,*^19^). Although ^123^I-FP-CIT SPECT is used in most hospitals as a diagnostic tool to support or exclude dopaminergic degeneration in routine practice, some institutes do use ^18^F-FDOPA PET for this purpose (35). Like DAT imaging, ^18^F-FDOPA PET is a sensitive technique, but a recent metaanalysis showed that the loss of striatal AADC activity is consistently smaller than that of striatal DAT activity in PD (5), possibly because of upregulation of AADC activity in surviving monoaminergic neurons. Consequently, especially in early stages of PD, ^18^F-FDOPA PET might be less sensitive to detect the dopaminergic deficit, but this postulate has not been proven yet (36).

#### VMAT-2 Imaging

Although the number of studies on VMAT-2 PET imaging in neurodegenerative disorders such as PD is much smaller than the number of studies on DAT imaging and ^18^F-FDOPA, the above-mentioned patterns of loss of striatal binding do—generally speaking—match the findings of DAT studies (37–40). Also, a 2018 study showed that the diagnostic accuracy of VMAT-2 imaging is high in patients with CUPS (41).

### Imaging of Postsynaptic Striatal Dopaminergic D_2/3_ Receptors

Dopamine receptors can be differentiated in dopamine D_1_- and D_2_-like receptors. D_1_ and D_5_ receptors belong to the group of D_1_-like receptors, whereas D_2_, D_3_, and D_4_ receptors belong to the group of D_2_-like receptors. Importantly, most radiopharmaceuticals used in PET/SPECT studies are nonselective tracers and bind to D_1/5_ or D_2/3_ receptors (Fig. 1) (6*,*^35^).

Dopamine D_2_ receptors are expressed presynaptically in dopaminergic neurons. These receptors are called autoreceptors and play a role in the regulation of dopamine release (Fig. 1). Dopamine D_2/3_ receptors, located in the striatum, are expressed predominantly postsynaptically (6).

MSA-P and PSP are characterized not only neuropathologically by degeneration of nigrostriatal dopaminergic neurons but also by loss of striatal D_2_-like receptors (42*,*^43^). PET and SPECT studies showed loss of postsynaptic dopamine D_2/3_ receptors in PSP and MSA-P (44). For many years, radiotracers such as ^123^I-IBZM or ^11^C-raclopride (Fig. 1) have been used in clinical practice to differentiate PSP/MSA-P from PD. Importantly, a recent metaanalysis of dopamine D_2/3_ receptor studies in PD showed increased D_2/3_ receptor binding (particularly on the contralateral side) in an early state of PD compared with control values (probably reflecting upregulation), but after a disease duration of approximately 4 y, PD patients had lower striatal D_2/3_ receptor binding values than did controls (probably due to downregulation). This metaanalysis also showed that PSP and MSA-P patients indeed had lower striatal dopamine D_2/3_ receptor binding than in PD, but this loss was only around 14% and 22%, respectively (44). This result indicates that imaging findings are in line with autopsy findings but also that the intraindividual values for striatal dopamine D_2/3_ receptor binding do show a clear overlap between PD and MSA-P or PSP. Although striatal D_2/3_ receptor binding has been used for many years in routine practice, in light of these diagnostic uncertainties, the diagnostic use of dopamine D_2/3_ receptor imaging is not recommended anymore. Interestingly, the use of ^18^F-FDG PET is probably superior to dopamine D_2/3_ receptor binding in differentiating PD from MSA-P or PSP and may be used in clinical practice for diagnostic support (44–47).

## IMAGING DOPAMINERGIC NEUROTRANSMISSION IN NEURODEGENERATIVE DISORDERS IN A RESEARCH SETTING

In the past 2 decades, an important research topic, related to imaging of the presynaptic pathway, has been the detection of nigrostriatal dopaminergic degeneration in the preclinical phase of neurodegenerative disorders. This topic is relevant, not only from a scientific point of view (e.g., to examine how many years before the motor signs of PD the nigrostriatal degeneration starts) but also to examine whether molecular imaging is able to detect subjects in the preclinical phase of neurodegeneration and, if so, to determine the extent of degeneration. This ability is relevant, because when the motor signs of PD start, approximately half of DAT expression in the putamen is already lost (5), potentially hampering a potentially successful intervention aimed to slow disease progression. Fortunately, molecular imaging studies showed the ability to detect nigrostriatal degeneration in subjects with rapid-eye-movement sleep behavior disorder (RBD), hyposmia, and late-onset depression, all of which are related to an increased risk for developing a movement disorder characterized by a dopaminergic deficit (7–10).

There is consensus among PD researchers that disease-modifying drugs and neuroprotective drugs are likely to be most effective at an early stage of PD, when delaying disease progression will be most effective (16). At this stage, a large number of the nigrostriatal dopaminergic neurons are already lost (5). Also, at this stage it can be especially difficult to diagnose PD clinically (15*,*^16^). Since DAT imaging is a sensitive imaging tool to detect PD in an early disease stage (11) and there is consensus that subjects having scans without evidence of dopaminergic deficit show a much slower motor deterioration than subjects whose scans do show dopaminergic degeneration (33*,*^48^), the European Medicines Agency has qualified DAT imaging as an enrichment biomarker for clinical trials targeting early stages of PD (i.e., within 1–2 y of clinical diagnosis) (16). Data for the large Parkinson Research Examination of CEP-1347 study and the Parkinson Progression Markers Initiative study were essential to reach this important milestone (33*,*^49^*,*^50^). The first application of DAT imaging as an enrichment biomarker has been published recently (51). It is likely that DAT imaging will be increasingly used in clinical trials that evaluate the efficacy of potential drug-modifying drugs in early PD.

The Parkinson Progression Markers Initiative data are publicly available, offering the unique opportunity for all interested research groups to perform analyses on this large dataset. For example, studies using this dataset have been performed to test the relationship between striatal DAT binding and cognitive executive impairment in PD, as well as on the relationship between DAT binding and α-synuclein in the cerebral spinal fluid (52*,*^53^).

It is now well accepted that neurodegenerative diseases such as PD, MSA, and PSP are not single disease entities (54*,*^55^). In this regard, it is of interest that molecular imaging studies showed the loss of striatal DAT binding to be more pronounced in the akinetic-rigid subtype than in the tremor-dominant subtype (56). Also, striatal DAT binding may be higher in women than men with PD, at symptom onset and throughout the course of PD, as is in line with the observation that women more often present with tremor than do men (57). These findings suggest a more benign phenotype in women with PD (57). Interestingly, Horsager et al. recently proposed that PD may comprise 2 subtypes: brain-first versus body-first (58). They postulated that in the brain-first subtype, degeneration starts in a single hemisphere, leading to asymmetric nigrostriatal degeneration, whereas in the body-first form, the initial enteric pathology will spread through vagal innervation, leading to a more symmetric degeneration. Indeed, in line with their postulation, a recent combined study of ^18^F-FDOPA PET and ^123^I-FP-CIT SPECT on isolated RBD (which is suggested to be the prototype of the body-first subtype) and on de novo PD patients with and without RBD showed a more symmetric degeneration in isolated RBD subjects than in PD patients without RBD (59). Finally, DAT binding is lower in MSA-P than in MSA-C (60).

The SPECT tracer ^123^I-FP-CIT is not a selective DAT tracer, as this radiotracer also shows a modest affinity for the serotonin transporter. Studies on healthy controls showed that it is actually extrastriatal, but not striatal, ^123^I-FP-CIT binding that can be blocked by a selective serotonin reuptake inhibitor (61*,*^62^). Previous work has shown that analyses of extrastriatal ^123^I-FP-CIT binding may contribute to the differential diagnosis of parkinsonian syndromes, in that not only striatal DAT binding is lower in PSP and MSA-P than in PD but also extrastriatal binding may be lower in some brain areas such as the diencephalon (21*,*^63^). Also, DLB patients may show lower ^123^I-FP-CIT binding in the thalamus than do PD patients (64).

Selective DAT tracers have been developed successfully (62*,*^65^); one example is ^18^F-FE-PE2I (Fig. 1). As expected, ^18^F-FE-PE2I PET studies showed the ability to detect the loss of striatal DAT binding in PD (66), and small head-to-head studies with ^123^I-FP-CIT SPECT showed that ^18^F-FE-PE2I is not inferior to ^123^I-FP-CIT in detecting striatal DAT loss (65*,*^67^). However, it is still unclear whether this PET tracer will replace ^123^I-FP-CIT as a diagnostic tool in the future.

SPECT and PET tracers for dopamine D_2/3_ receptors (Fig. 1) can be used not only to assess the baseline in vivo availability of these receptors but also to assess endogenous dopamine release (displacement experiments). Many studies have been performed to assess the baseline availability of these receptors in vivo in PD (44*,*^68^). Generally speaking, these studies showed an upregulation of striatal dopamine D_2/3_ receptors in early PD, likely a compensating effect on the presynaptic dopaminergic deficit, that faded when the disease duration increased (44). Using the dopamine release paradigm, Piccini et al. showed that a methamphetamine challenge was able to induce a detectable dopamine release in the putamen of advanced PD cases, although this release was much lower than in healthy controls (69). Also, impulse control disorders are common in PD, and impulse control disorders in PD are associated with relatively increased dopamine in the ventral striatum (70). Interestingly, a ^11^C-raclopride PET study showed that although striatal dopamine D_2/3_ binding is similar at baseline between PD patients with and those without impulse control disorders, dopamine release after presentation of reward-related visual cues (and after a levodopa challenge) was higher in PD patients with impulse control disorders (71). Finally, dopamine release is also assessed to better understand motor fluctuation in PD. Using ^11^C-raclopride PET, de la Fuente-Fernández et al. showed that 1 h after a levodopa challenge, the dopamine levels were increased in the putamen of PD patients with motor fluctuations as compared with those without such fluctuations (72). All in all, these findings highlight that disturbance of the dopaminergic system in neurodegenerative disorders is sometimes detectable only when the dopaminergic system is challenged.

The number of studies on dopamine D_1/5_ receptors, using tracers such as ^11^C-SCH 23390 (Fig. 1), is much lower than the number on D_2/3_ receptors. In general, these studies have not shown a significant difference in striatal D_1_ receptor binding between PD and controls (68).

**FIGURE 4.**
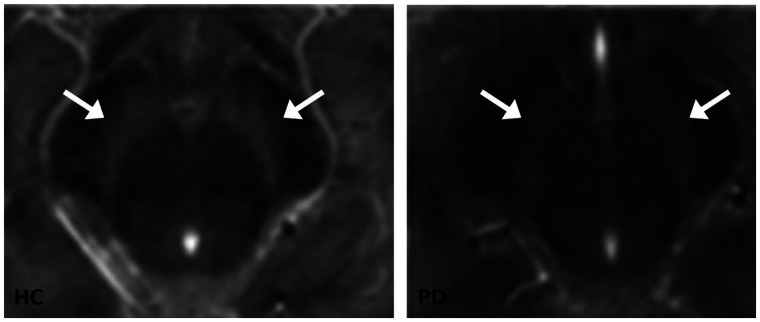
Transversal images of neuromelanin-sensitive MRI scans of mesencephalon. Substantia nigra is visible as hyperintense area next to cerebral peduncles. Left panel shows example of neuromelanin-sensitive MRI in healthy control, and right panel shows example of patient with PD. Loss of signal can be seen in substantia nigra in PD patient. Arrows point to substantia nigra. (Reprinted from (74).)

Different aspect of the dopaminergic system can be assessed directly only by imaging techniques such as PET or SPECT. However, novel MRI sequences are capable to indirectly assess the dopaminergic system in vivo. The so-called neuromelanin-sensitive MRI is capable of visualizing the loss of neuromelanin-containing dopaminergic cells in the substantia nigra (73*,*^74^). Recently, the correlation between signal intensity on neuromelanin-sensitive MRI and neuromelanin concentration in the substantia nigra was elegantly proven by an autopsy study by Cassidy et al. (75). Indeed, many studies have shown that the neuromelanin signal in the substantia nigra is lower in PD, MSA, and PSP than in controls (Fig. 4) (76*,*^77^). Although this new technique is promising, with potential advantages over DAT imaging (e.g., lower costs, faster acquisitions), large prospective clinical studies on patients with CUPS, and studies on CUPS patients with autopsy conformation, are needed to assess its diagnostic power in clinical practice.

## CONCLUSION

Imaging of the nigrostriatal pathway has high diagnostic accuracy in detecting nigrostriatal degeneration in common movement disorders characterized by a presynaptic dopaminergic deficit and in patients with CUPS. In clinical practice, imaging of striatal dopamine D_2/3_ receptors no longer plays a major diagnostic role in the differential diagnosis of parkinsonian disorders. Regarding research, imaging of the dopaminergic system plays a major role in, for example, examining nigrostriatal degeneration in preclinical and premotor stages of neurodegenerative disorders or motor complications in the treatment of PD. Finally, neuromelanin-sensitive MRI is a promising new tool to study nigrostriatal degeneration in vivo.

## DISCLOSURE

Jan Booij is consultant at GE Healthcare and received research grants from GE Healthcare (all payments to the institution). Rob de Bie received research grants from GE Healthcare (paid to the institution). No other potential conflict of interest relevant to this article was reported.
